# Short- and Mid-Term Impacts of COVID-19 Outbreak on the Nutritional Quality and Environmental Impact of Diet

**DOI:** 10.3389/fnut.2022.838351

**Published:** 2022-03-11

**Authors:** Lucile Marty, Blandine de Lauzon-Guillain, Sophie Nicklaus

**Affiliations:** ^1^Centre des Sciences Du Goût et de l'Alimentation, CNRS, INRAE, Institut Agro Dijon, Université Bourgogne Franche-Comté, Dijon, France; ^2^Université Paris Cité, CRESS, INSERM, INRAE, Paris, France

**Keywords:** COVID-19, diet, food choice motives, nutritional quality, environmental impact, longitudinal study

## Abstract

**Background:**

Changes in dietary behaviors that occurred at the beginning of the COVID-19 outbreak and in particular during the first national lockdowns have been extensively studied across countries. Beyond the understanding of contextual changes in diets due to a temporary lockdown, it is of interest to study longer-term consequences of the COVID-19 outbreak as sustained changes in diets may have both an impact on population health and the environment.

**Objectives:**

This study aimed to examine both short- (after 1 month) and mid-term (after 1 year) impacts of the COVID-19 outbreak on the nutritional quality and environmental impact of diets, and as a secondary objective on food choice motives.

**Methods:**

We collected dietary data [food frequency questionnaire (FFQ)] and the importance of nine food choice motives through online questionnaires before, during, and after 1 year of the first lockdown for 524 French participants. Adherence to the French dietary recommendations was estimated using the simplified PNNS-GS2, which scores from −17 to 11.5. Environmental impact of diets was assessed by calculating greenhouse gas emissions in CO2eq/2,000 kcal.

**Results:**

We showed a short-term decrease in nutritional quality (−0.26 points on sPNNS-GS2, *p* = 0.017) and environmental impact (−0.17 kg CO2eq/2,000 kcal, *p* = 0.004) but this decrease was only temporary, and nutritional quality (−0.01 points on sPNNS-GS2, *p* = 0.974) and environmental impact (−0.04 kg CO2eq/2,000 kcal, *p* = 0.472) were not different from their initial values 1 year later. Some of the food choice motives followed the trend of a short-term increase and a mid-term stability (health, natural content, ethical concern, and weight control). On the contrary, we showed both short- and mid-term decreases in convenience, familiarity, and price motives.

**Conclusion:**

Changes in diets and motives observed during the first lockdown were mostly temporary. However, we highlighted a sustained decrease in the importance of perceived constraints due to food shopping and food preparation which may suggest a trend toward a more positive perception of food-related activities.

## Introduction

The COVID-19 pandemic and the restrictions that have been imposed by the governments to avoid transmission of the virus (e.g., nationwide lockdowns) led to changes in dietary habits (1). However, most studies that have been published so far examined short-term impacts of the first lockdown on eating behaviors across countries (2–4) and longer-term impacts of the COVID-19 outbreak on dietary habits remain to be explored. Yet, as suboptimal intakes of several foods and nutrients are the major risk factors for non-communicable diseases (5), changes in dietary behaviors sustained over a long period of time may affect the health status of the population.

Only few studies recorded detailed food consumption and compared the nutritional quality of the overall diet before and during the COVID-19 outbreak and they led to mixed results: an increase in the nutritional quality in Quebec (6), a decrease in Spain (7) and Burkina Faso, Ethiopia and Nigeria (8), no change in France but with a large interindividual variability in dietary changes (9). These results suggest that eating habits may have changed in both favorable and unfavorable directions in association with context and individual characteristics. In a previous study investigating the nutritional quality of diet and food choice motives during the first lockdown in France, we also highlighted heterogeneity in changes (10). We showed a decrease in the nutritional quality of diet for individuals who comforted themselves with food during the lockdown (48%) but an increase in the nutritional quality for individuals who tried to better control their weight during the lockdown (29%). In addition, in this same previous study, we observed an increase in ethical concern (21% of the participants) and natural content (19%) as food choice motives during the lockdown, which suggests a growing awareness of the importance of the environmental impact of food choices (10). These results echoed the raise in environmental awareness during the COVID-19 outbreak that has been documented and described by numerous scientists (11–15). However, to our knowledge, the impact of the COVID-19 outbreak on the environmental impact of diets has never been studied.

In cross-sectional studies, higher motives related to health, natural content, and ethical concern have been associated with higher nutritional quality of diet (16–18) and with higher consumption of organic food products (19) whereas higher motives related to price, familiarity, and convenience have been associated with lower nutritional quality of diet (16, 18) and lower consumption of organic food products (19). Nonetheless, less is known about how changes in food choice motives at an individual level may influence food consumption, which would provide valuable insights to identify food choice motives to be tackled through public health interventions to increase the nutritional quality or decrease the environmental impact of diets. As major life disruptions may lead the individuals to engage in a new process of food choice decision-making and thus to reconsider their food choice motives (20, 21), the COVID-19 outbreak gave the unique opportunity to investigate how changes in food choice motives may influence the nutritional quality and the environmental impact of diet at an individual level. In a previous study, we found that short-term changes in mood motives and weight control motives impacted the nutritional quality of diet, negatively and positively, respectively (10). Surprisingly, short-term changes in health motives did not translate into an increased nutritional quality (10). Because dietary habits might be hard to change even though food choice motives have been reconsidered (22), we hypothesized that longer-term changes in food choice motives may be more impactful. For instance, changes in health or ethical concern motives may have an impact on the nutritional quality and environmental impact of the diet after 1 year, whereas these changes may have no impact after 1 month.

This study aimed to examine short- (after 1 month) and mid-term (after 1 year) impacts of the COVID-19 outbreak on both the nutritional quality and the environmental impact of diets. This study adds to a previous investigation of changes in nutritional quality during the first lockdown in France (10) by also analyzing the evolution of greenhouse gas emissions of diets. As secondary objectives, we aimed to examine short- and mid-term impacts of the COVID-19 outbreak on organic and local food consumption and on food choice motives. As exploratory objectives, we examined the association between short- and mid-term changes in food choice motives and short- and mid-term changes in the nutritional quality and environmental impact of diets.

## Materials and Methods

### Study Design

This was a longitudinal, preregistered (https://osf.io/gwfdb/) online survey using Qualtrics platform (www.qualtrics.com) following up a survey on nutritional quality and food choice motives before and during the first lockdown in France conducted in spring 2020; results have been published elsewhere (10). In the 2020 survey, we retrospectively recorded at the same time dietary data and food choice motives for 938 individuals during the month before the first lockdown (from 17 February to 16 March 2020) and during the first month of lockdown (from 17 March to 16 April 2020). A number of 938 individuals who took part in the 2020 survey were emailed exactly 1 year later, that is on 2 May, 2021. They were asked to answer the same set of questionnaires as in 2020 regarding the 1-month period from the 1 to 30 April 2021: a food frequency questionnaire (FFQ), a food choice motives questionnaire, and a questionnaire on organic and local food consumption. We thus recorded longitudinal data on three 1-month periods per individual: before the first lockdown, during the first lockdown, and 1 year after the first lockdown.

### Data Collection

Data were collected from 2 to 15 May to 2021. The participants were all registered in the Chemosens Platform's PanelSens database declared to the relevant authority (Commission Nationale Informatique et Libertés; CNIL; n°1148039). Eligible participants took part in the 2020 survey and had not moved since then. All participants were contacted by email and provided consent for their participation after being informed that the purpose of the study was to investigate food choices 1 year after the first lockdown. They received a 10 € Amazon voucher in return for their participation. As in the 2020 survey, three attention check questions (e.g., “How many times have you visited the planet Mars?”) were included in various parts of the questionnaire. The study was approved by the relevant ethical evaluation committee for research (reference: Comité d'Evaluation Ethique de l'Inserm n°20-683bis, delivered on 30 March 2021). All the questionnaires (in French) are available on the OSF page of the project (https://osf.io/gwfdb/).

### Food Frequency Questionnaire

Participants reported their diet for the previous month using a validated FFQ that includes 109 foods, 12 non-alcoholic drinks, and 4 alcoholic drinks with frequency assessed by a 6-item scale from “never” to “several times a day” (23). Food groups included in the FFQ were as follows: bread and cereals (4 items), raw vegetables (4 items), cooked vegetables (13 items), starchy foods (10 items), meat and eggs (15 items), fish (9 items), mixed dishes (12 items), dairy products (10 items), fruit (11 items), biscuits and cakes (14 items), and sauces (7 items). Usual portion sizes were estimated with the photographs for different food types on a 5-level scale, derived from the SU.VI.MAX portion book (24) or using average portion size. The consumption frequency of each item was transformed into daily frequency, and daily intake was calculated by multiplying the daily frequency by the estimated portion size. Individual nutrient intakes were calculated by multiplying the daily intake of each food item by its nutritional values from the SU.VI.MAX nutrient composition database (25).

Adherence to the French dietary recommendations before, during, and 1 year after the first lockdown was evaluated using the simplified PNNS-GS2 (sPNNS-GS2), an index previously designed by Chaltiel et al. (26) to reflect adherence to the main 2017 French dietary recommendations. The sPNNS-GS2 builds on the distinction between malus components (less healthy food groups which consumption should be limited, carrying a negative score, i.e., red meat, processed meat, sugary foods, sweet-tasting beverages, alcoholic beverages, and salt) and bonus components (healthier food groups carrying a positive score, i.e., fruits and vegetables, nuts, legumes, whole-grain food, milk and dairy products, fish, and seafood). The sPNNS-GS2 was computed for each participant with slight modifications to the calculation (range: 17–11.5). The sPNNS-GS2 originally included bonus points for added fat below 16% of energy intake (26). The FFQ did not make it possible to calculate the percentage of energy intake accounted for added fat, especially added oils, and this component was not included in the score calculation, as in Marty et al. (10). We also calculated short-term Δ nutritional quality and mid-term Δ nutritional quality, as the difference in sPNNS-GS2 between during and before the first lockdown and between 1 year later and before the first lockdown, respectively. Δ nutritional quality > 0 indicated an increase in nutritional quality over time.

Greenhouse gas emissions (GHGE) in kg CO2eq/kg of the individual diets were calculated before, during, and 1 year after the first lockdown as an indicator of the environmental impact of the diet (27). GHGE were derived from the French food environmental impact database Agribalyse 3.0 drawn up by the French Agency for Ecological Transition that includes GHGE values for 2,480 common food items based on Life Cycle Analyses of food products (28). The items of the FFQ were associated with all the corresponding food items from Agribalyse 3.0. GHGE of each item of the FFQ were calculated as the average GHGE of individual foods from Agribalyse 3.0 associated with each item. GHGE of participants' daily diets were calculated by multiplying the daily intake of each food item by its associated GHGE per kg. We also calculated short-term Δ GHGE and mid-term Δ GHGE, as the difference in GHGE of diets between during and before the first lockdown and between 1 year later and before the first lockdown, respectively. Δ GHGE > 0 indicated an increase in GHGE over time.

### Organic and Local Food Consumption Questionnaire

Participants answered questions about their consumption frequency of organic and local food products for 12 food categories on a 3-point scale: 2 = most of the time, 1 = occasionally, and 0 = never. The 12 categories were as follows: fruit, vegetables, dairy products, meat and fish, eggs, grains, bread, oil, ready-to-eat meals, biscuits, tea and coffee, and wine and beers. This questionnaire was adapted from a previously published version that assessed organic food consumption only and included six additional food or non-food product categories (29); we excluded the six additional categories because they were not part of the FFQ. Organic consumption scores and local consumption scores were calculated before, during, and 1 year after the first lockdown as the mean of participants' responses across the 12 food categories.

### Food Choice Motives Questionnaire

Food choice motives were assessed using a French version of the Food Choice Questionnaire developed in English by Steptoe et al. (30) and adapted by Cottet et al. (31). The French version included 24 items and nine subscales: health (3 items), convenience (3 items), sensory appeal (3 items), natural content (3 items), ethical concern (2 items), weight control (3 items), mood (3 items), familiarity (2 items), and price (2 items). For each subscale, scores before, during, and 1 year after the first lockdown were computed by averaging ratings for individual items. The scores ranged from 1 to 4: 1 = not at all important; 2 = a little important; 3 = moderately important; 4 = very important. Short-term Δ motives and mid-term Δ motives were calculated as the difference of the score for each of the nine subscales between during and before the first lockdown and between 1 year later and before the first lockdown, respectively. Δ motives > 0 indicated an increased importance of the motives over time.

### Sociodemographic Questionnaire

Participants were asked for their gender, employment status, highest educational qualification, dietary restriction (none, vegetarian, vegan, gluten-free, sugar-free, lactose-free, allergies), dieting status (yes or no), and weight and height at the time of completion of the initial 2020 online survey. Self-reported body mass index (BMI) was calculated in kg/m^2^. In 2021, they were asked about their perceived change in eating habits (yes a lot, yes moderately, yes a bit, and no) and in time spent cooking (7-point scale from increased a lot to decreased a lot) during the pandemic. COVID-19-related questions (being or having been ill) were also included for descriptive purpose.

### Statistical Analyses

We followed an analytic plan that was preregistered prior to 2021 data collection (https://osf.io/gwfdb/). Any deviations from the preregistered analytic plan are described in Supplementary Table S1. Only participants who completed the 2020 and 2021 surveys were included in the analyses. Participants who failed at least one attention check were excluded. We analyzed data from participants who reported plausible energy intake before, during, and 1 year after the first lockdown, that is, ≥500 and ≤ 3,500 kcal/ day for women, and ≥800 and ≤ 4,000 kcal/day for men (32, 33). We compared participants that were included and excluded from the present analyses based on sociodemographic measures and dietary outcomes (2020 data).

Cronbach's α were calculated for the organic and local food consumption questionnaire (before, during, and after 1 year, respectively): organic score (0.91; 0.90; 0.89) and local score (0.87; 0.88; 0.85). Cronbach's α were also calculated for each food choice motive (before, during, and after 1 year, respectively): health (0.70; 0.71; 0.74), convenience (0.89; 0.85; 0.88), sensory appeal (0.65; 0.66; 0.64), natural content (0.87; 0.86; 0.85), ethical concern (0.64; 0.63; 0.69), weight control (0.81; 0.84; 0.83), mood (0.64; 0.64; 0.66), familiarity (0.63; 0.62; 0.76), and price (0.58; 0.63; 0.66).

For primary analyses examining the effect of the COVID-19 outbreak on the nutritional quality and environmental impact of diet, linear mixed models were used to test the effect of time (categorical: before, during, and after 1 year of the first lockdown) on sPNNS-GS2 and GHGE, with random effect of participant to account for correlation between repeated measures. For secondary analyses examining the effect of the COVID-19 outbreak on organic and local food consumption, linear mixed models were used to test the effect of time on organic and local consumption scores, with random effect of participant to account for correlation between repeated measures. For secondary analyses examining the effect of the COVID-19 outbreak on food choice motives, linear mixed models were used to test the effect of time on the nine subscales of the Food Choice Questionnaire, with random effect of participant to account for correlation between repeated measures. All linear mixed models were replicated controlling for age, gender, educational level, and declared initial BMI. Pairwise adjusted mean comparisons were conducted for all raw and adjusted models.

Exploratory analyses aimed to examine the influence of short- and mid-term changes in food choice motives on short- and mid-term changes in the nutritional quality and environmental impact of diet, respectively. Four multiple linear regressions testing the effect of Δ motives on Δ nutritional quality and Δ GHGE at both short- and mid-term were run. They were also replicated controlling for age, gender, educational level, and declared initial BMI.

All statistical analyses were performed using SAS version 9.4 (SAS Institute, Inc. 2013 SAS^®^ 9.4. Cary, NC). The level of significance was set at *p* < 0.05 for preregistered primary, secondary, and exploratory analyses.

## Results

### Participants

A number of 938 participants who took part to the 2020 survey were contacted by email and 594 consented to participate. We excluded 18 participants who did not complete the study, 17 who failed an attention check, 34 who reported implausible energy intake, and one who reported an incorrect identification number resulting in a sample size of 524 participants with correct responses before, during, and 1 year after the first lockdown. Table 1 presents participants' characteristics. The participants in the 2021 study were slightly older than the participants in the 2020 study who did not complete the 2021 survey (Supplementary Table S2) but did not differ regarding gender, employment status, highest educational qualification, initial BMI, and dietary outcomes before the first lockdown (energy intake, sPNNS-GS2, and GHGE). In 2021, 72% of the participants declared that their eating habits have changed since the beginning of the pandemic and 96% reported an increase in time spent cooking. Moreover, 86 participants (17%) declared that they have been affected by COVID-19 since the beginning of the pandemic.

**Table 1:** Participants' characteristics in 2020, *n* = 524.

**Age, years, mean (SD)**	39.5 (12.0)
**Gender, female, ***n*** (%)**	417 (79.6)
**Employment status, ***n*** (%)**
Full or part-time	406 (77.4)
Student	34 (6.5)
Retired	33 (6.3)
Looking for a job	37 (7.1)
Looking after home	5 (1.0)
Other	9 (1.7)
**Highest educational qualification, ***n*** (%)**
< High-school +2 years diploma	129 (24.6)
High-school +2 years diploma	107 (20.4)
High-school +3 or +4 years diploma	131 (25.0)
≥High-school +5 years diploma	157 (30.0)
**Dietary restrictions, none, ***n*** (%)**	468 (89.3)
**Dieting status, ***yes***, ***n*** (%)**	74 (14.1)
**Reported BMI, kg/m^2^, mean (SD)**	24.4 (4.9)
Implausible weight or height*, *n* (%)	6 (1.1)

* *Excluding weight <30 or >250 kg, height <1.45 or >3 m*.

### Evolution of Nutritional Quality, Environmental Impact, Organic and Local Food Consumption, and Food Choice Motives

A significant effect of time was found on nutritional quality and GHGE of diet and on organic and local food consumption (Table 2). Total energy intake and GHGE per day increased on the short-term then remained constant. We observed a short-term decrease but no mid-term changes in nutritional quality and GHGE per 2,000 kcal of diet whereas the increase in organic and local food consumption was mid-term only. A significant effect of time was also found on all food choice motives, except for sensory appeal motives that remained stable over time (Table 2). Regarding the evolution of food choice motives over time, we observed a short-term increase in health, natural content, ethical concern, and weight control motives but no mid-term increase and even an overall decrease compared to before the first lockdown for ethical concern motives. On the contrary, the decrease in convenience, familiarity, and price motives was both short-term and mid-term as was the increase in mood motives. The results were the same in both raw and adjusted models.

**Table 2:** Nutritional quality, environmental impact, organic and local food consumption, and food choice motives over time.

	**Before first**	**During first**	**1 year after first**	**Linear mixed models** *			
	**lockdown**	**lockdown**	**lockdown**	**Raw (*****n*** **= 524)**		**Adjusted^†^** **(*****n*** **= 518)**	
				***F***	***p***	***F***	***p***
Total energy (kcal/d)	1,667 (581) a	1,888 (632) b	1,871 (635) b	**45.63**	**<0.001**	**45.57**	**<0.001**
sPNNS-GS2	1.14 (2.49) a	0.88 (2.69) b	1.15 (2.58) a	**3.81**	**0.022**	**3.73**	**0.024**
GHGE (kg CO_2_eq/d)	4.72 (2.05) a	5.20 (2.23) b	5.22 (2.12) b	**22.41**	**<0.001**	**23.37**	**<0.001**
GHGE (kg CO_2_eq/2,000 kcal)	5.67 (1.46) a	5.50 (1.40) b	5.63 (1.45) a	**4.42**	**0.012**	**4.92**	**0.008**
Organic score	0.63 (0.49) a	0.62 (0.48) a	0.68 (0.47) b	**12.99**	**<0.001**	**13.68**	**<0.001**
Local score	0.60 (0.42) a	0.61 (0.45) ab	0.64 (0.41) b	**4.40**	**0.013**	**4.42**	**0.012**
*Food choice motives*							
Health	2.75 (0.68) a	2.86 (0.70) b	2.70 (0.69) a	**21.45**	**<0.001**	**20.76**	**<0.001**
Convenience	2.49 (0.83) a	2.07 (0.78) b	2.40 (0.79) c	**76.48**	**<0.001**	**74.95**	**<0.001**
Sensory appeal	3.31 (0.53) a	3.33 (0.55) a	3.31 (0.52) a	0.82	0.443	0.68	0.505
Natural content	2.89 (0.78) a	2.94 (0.78) b	2.87 (0.75) a	**4.59**	**0.010**	**4.57**	**0.011**
Ethical concern	2.82 (0.80) a	2.88 (0.81) b	2.73 (0.83) c	**15.78**	**<0.001**	**15.68**	**<0.001**
Weight control	2.30 (0.71) a	2.42 (0.78) b	2.29 (0.75) a	**13.19**	**<0.001**	**12.85**	**<0.001**
Mood	2.19 (0.70) a	2.44 (0.76) b	2.32 (0.71) c	**39.11**	**<0.001**	**39.12**	**<0.001**
Familiarity	2.55 (0.73) a	2.45 (0.78) b	2.30 (0.77) c	**30.64**	**<0.001**	**30.47**	**<0.001**
Price	2.83 (0.61) a	2.78 (0.70) ab	2.74 (0.66) b	**6.02**	**0.003**	**6.04**	**0.003**

*All values are mean (SD). Values with the same letter are not statistically different at α = 0.05, pairwise comparisons for raw and adjusted models led to the same results*.

* *Linear mixed models testing the effect of time on dependant variables*.

† *Control variables: age, gender, initial BMI (six missing values), and highest educational qualification*.

*In bold: significant changes over time at α = 0.05*.

### Associations Between Short- and Mid-Term Changes in Food Choice Motives and Short- and Mid-Term Changes in Nutritional Quality or Environmental Impact of Diet

As exploratory analyses, we examined the associations between short- and mid-term changes in food choice motives and short- and mid-term changes in nutritional quality and environmental impact (GHGE/2,000 kcal) of diet (Table 3). Short- and mid-term increases in weight control motives were associated with short- and mid-term increases in nutritional quality, respectively, and also with short- and mid-term increases in GHGE/2,000 kcal. On the contrary, short-term increase in price motives was associated with short-term decrease in nutritional quality, and with short-term decrease in GHGE/2,000 kcal. Mid-term increase in natural content motives was associated with mid-term increase in nutritional quality only. Short-term increase in mood motives was associated with short-term increase in GHGE/2,000 kcal. On the contrary, mid-term increase in ethical concern motives was associated with mid-term decrease in GHGE/2,000 kcal.

**Table 3:** Influence of Δ motives on Δ nutritional quality and Δ GHGE/2,000 kcal, short-term (during–before lockdown) and mid-term (one year later–before lockdown).

	Dependant variable*: Δ nutritional quality				Dependant variable*: Δ GHGE/2,000 kcal			
	**Δ short-term^‡^**		**Δ mid-term^‡^**		**Δ short-term^‡^**		**Δ mid-term^‡^**	
	**Raw model** **(***n*** = 524)**	**Adjusted model^†^ (*n* = 518)**	**Raw model** **(***n*** = 524)**	**Adjusted model^†^ (*n* = 518)**	**Raw model** **(***n*** = 524)**	**Adjusted model^†^ (*n* = 518)**	**Raw model** **(***n*** = 524)**	**Adjusted model^†^ (*n* = 518)**
	**β (95%IC)**	**β (95%IC)**	**β (95%IC)**	**β (95%IC)**	**β (95%IC)**	**β (95%IC)**	**β (95%IC)**	**β (95%IC)**
Δ Health	0.05 (−0.59; 0.70)	0.05 (−0.60; 0.70)	0.06 (−0.33; 0.45)	0.03 (−0.37; 0.42)	−0.03 (−0.35; 0.30)	−0.01 (−0.34; 0.31)	0.07 (−0.15; 0.29)	0.09 (−0.13; 0.31)
Δ Convenience	0.19 (−0.08; 0.46)	0.20 (−0.08; 0.48)	−0.03 (−0.29; 0.23)	−0.04 (−0.30; 0.22)	−0.01 (−0.15; 0.13)	−0.01 (−0.15; 0.13)	−0.08 (−0.22; 0.07)	−0.06 (−0.21; 0.08)
Δ Sensory appeal	−0.27 (−1.09; 0.54)	−0.33 (−1.16; 0.49)	−0.39 (−0.79; 0.02)	−0.38 (−0.78; 0.03)	−0.09 (−0.50; 0.32)	−0.11 (−0.53; 0.30)	−0.12 (−0.35; 0.10)	−0.14 (−0.36; 0.09)
Δ Natural content	0.34 (−0.38; 1.07)	0.37 (−0.36; 1.11)	**0.42** (0.04; 0.80)	**0.44** (0.05; 0.83)	−0.04 (−0.41; 0.32)	−0.01 (−0.38; 0.35)	0.19 (−0.02; 0.41)	0.21 (−0.01; 0.42)
Δ Ethical concern	−0.10 (−0.62; 0.41)	−0.09 (−0.61; 0.43)	0.02 (−0.32; 0.36)	0.03 (−0.32; 0.37)	0.11 (−0.15; 0.37)	0.12 (−0.15; 0.38)	**−0.24** (−0.44; −0.05)	**−0.27** (−0.46; −0.08)
Δ Weight control	**0.99** (0.61; 1.36)	**0.98** (0.60; 1.36)	**0.42** (0.08; 0.76)	**0.42** (0.07; 0.76)	**0.21** (0.02; 0.40)	**0.20** (0.01; 0.40)	**0.23** (0.04; 0.43)	**0.22** (0.03; 0.41)
Δ Mood	−0.44 (−0.62; 0.03)	−0.47 (−0.94; 0.01)	−0.18 (−0.51; 0.14)	−0.21 (−0.54; 0.12)	**0.25** (0.01; 0.48)	**0.26** (0.03; 0.50)	0.15 (−0.03; 0.33)	0.14 (−0.05; 0.36)
Δ Familiarity	−0.07 (−0.46; 0.33)	−0.09 (−0.49; 0.30)	0.04 (−0.25; 0.33)	0.01 (−0.29; 0.30)	0.12 (−0.08; 0.32)	0.12 (−0.08; 0.32)	−0.11 (−0.27; 0.06)	−0.09 (−0.26; 0.07)
Δ Price	**−0.59** (−0.95; −0.22)	**−0.58** (−0.95; −0.21)	−0.11 (−0.48; 0.25)	−0.13 (−0.50; 0.24)	**−0.20** (−0.39; −0.02)	**−0.12** (−0.38; −0.01)	−0.09 (−0.29; 0.12)	−0.10 (−0.30; 0.11)

* *Multiple linear regressions testing the effect of the nine Δ motives simultaneously on dependant variables*.

† *Control variables: age, gender, initial BMI (six missing values), and highest educational qualification*.

‡ *Short- and mid-term changes in motives as predictors of short- and mid-term changes in dependant variables, respectively*.

*In bold: parameters significantly different from zero at α = 0.05*.

## Discussion

This longitudinal study explored individual changes in diet characteristics, namely nutritional quality, environmental impact, and organic and local food consumption, during the first year of the COVID-19 outbreak. We confirmed temporary decrease in nutritional quality and found temporary decrease in environmental impact per 2,000 kcal during the first lockdown but no sustained changes in the long run (which reflects no qualitative evolution of the diet). We found mid-term increase in total energy intake and in environmental impact per day (which reflects quantitative evolution of the diet). The initial life disruption caused by the lockdown had an impact on the quality of dietary patterns as it has been demonstrated in several studies (1). Here, we showed that it has been a time-limited adaptation to a contextual change rather than a reconsideration of food habits. However, we found a 1-year trend toward more organic and local food consumption consistent with the evolution of French food preferences over the past years (34, 35).

In line with the status quo regarding nutritional quality and environmental impact of diet after 1 year of pandemic, no mid-term changes were observed in health, natural content, ethical concern, and weight control motives. The increase in these motives during the first lockdown may have been incorrectly interpreted as a potentially lasting raise in health and environmental awareness (14). Nevertheless, we observed a sustained decrease over time in convenience, familiarity, and price motives. All the food choice motives that were associated with anticipated constraints due to food shopping (price), food preparation (convenience, familiarity), or food acceptability (familiarity) sustainably faded during the first year of pandemic. This observation echoes with 96% of the participants reporting an increase in the time they have spent cooking since the beginning of the pandemic. Several studies have reported an increase in home cooking during the first lockdown in France (10, 36, 37) explained by the increased number of meals taken at home due to the lockdown and by less time constraints due to the cessation of most out-of-home activities during this period. More importantly, this increase in time dedicated to food preparation and other social food-related activities (e.g., cooking and eating with family members) was perceived as positive and to be maintained after the first lockdown (38). In line with these previous observations, the sustained decrease in food choice motives related to anticipated constraints might denote a change in perception of food-related activities, from a chore to a social pleasurable time. In addition, it is noteworthy that sensory appeal motives remained stable and high, which is perhaps not surprising as the French population is known to primarily have pleasure-oriented attitudes toward food (39–41).

Previous studies have investigated the relationship between food choice motives and food choices across various samples of individuals and identified motivational profiles associated with specific food behaviors (42–44). However, cross-sectional associations do not predict what would happen in the case of a change in individual motivations over time. Here, we demonstrated that mid-term increase in motives related to natural content and weight control was related to an increased nutritional quality of diet and that mid-term increase in motives related to ethical concern was related to a decreased environmental impact of diet whereas a mid-term increase in weight control motive was related to an increased environmental impact of diet. However, contrary to what was observed in cross-sectional studies (16, 18), increased health and ethical concern motives were not associated with increased nutritional quality of diet. Our observations were limited to *natural* changes in food choice motives caused by the unusual situation of the COVID-19 outbreak and its consequences (e.g., successive lockdowns). Other life disruptions that result in longer-term contextual changes at individual levels may more likely result in sustained changes in food choice motives and consequently in food behaviors (21).

Collectively, the present results on food choice motives bring into question the conditions under which changes in motives may remain stable over time and translate into actual behavioral change. According to the COM-B model of behavior proposed by Michie et al. (45), motivation defined as all brain processes that energize and direct behavior plays a central role in predicting actual behavior. This model also depicts a retroactive loop from the behavior to the motivation; the repetition of a behavior in line with a motivation in turns reinforces this motivation. In our sample, a short-term increase in health motives was not associated with short-term changes in nutritional quality, that is, a behavior change that would have been expected in line with the change in motivation. One year later, health motives were back to their initial level. On the contrary, we observed both a short-term decrease in convenience motives and a short-term increase in time spent cooking during the first lockdown (10). One year later, convenience motives were still lower than before the first lockdown and participants declared having spent more time cooking during the past year. We may hypothesize that a change in motivation will be remaining only if it translates in a new behavior in line with the new motivation that successfully incorporates into a daily routine.

In this study, we recorded total dietary intake through a validated FFQ allowing to calculate indicators based on whole-diet composition. This is a major strength of this study as numerous studies that investigate the impact of the COVID-19 outbreak on food behaviors have only asked questions about the consumption of specific food groups, for example, healthy and unhealthy key food groups (4), sugared-sweetened beverages and sweet or salty snacks (2), and fresh food, non-perishable food, readymade meals, and sweet snacks (3). However, we note that FFQs are subjected to recall bias that may have affected dietary data. Another strength of this study is that the same questionnaires were completed three times by the same participants which makes this study the first to our knowledge that have investigated lasting effects of the COVID-19 outbreak on food behaviors. In addition, we took care of contacting the participants exactly 1 year after the first lockdown to record dietary intake at the same period of the year which limited seasonal effect on food consumption. Our sample was not representative of the French population and included more women and individuals with higher educational level, which constitutes a limitation of our study and limit the generalisability of the results. In addition, only 63% of the participants from the initial survey accepted to complete the follow-up survey which may have biased the sample toward individuals with the strongest interest in food. Finally, eight models were run to explore the associations between short- and mid-term changes in food choice motives and short- and mid-term changes in nutritional quality and environmental impact. In each model, we tested nine predictors, hence a total of 72 statistical tests which increased the likelihood of finding an association by chance alone.

## Conclusion

The slight decrease in nutritional quality and environmental impact of diet observed during the first lockdown was only temporary, and we observed no difference between before and 1 year after the beginning of the COVID-19 outbreak. In the same vein, after an increase during the first lockdown, motives related to health, natural content, and ethical concern went back to their initial level after 1 year of pandemic. On the contrary, motives related to convenience, familiarity, and price remained at a lower level of importance, which indicates a sustained decrease in perceived constraints due to food shopping and food preparation. It would be of interest to investigate how these changes would translate into new food habits in the long run, notably consumption of less convenient yet healthy and environment-friendly food items (e.g., pulses).

## Data Availability Statement

The original contributions presented in the study are publicly available. This data can be found here: https://osf.io/gwfdb/.

## Ethics Statement

The studies involving human participants were reviewed and approved by Comité d'Evaluation Ethique de l'Inserm, n°20-683bis. The patients/participants provided their written informed consent to participate in this study.

## Author Contributions

LM: conceptualization, investigation, formal analysis, and writing—original draft. BL-G: methodology, software, writing, reviewing, and editing. SN: conceptualization, investigation, writing, reviewing, and editing. All authors contributed to the article and approved the submitted version.

## Funding

This work was supported by grants from ANR (ANR-15-CE21-0014, PUNCH grant to SN), the Conseil Régional Bourgogne, Franche-Comte (PARI grant), and the FEDER (European Funding for Regional Economic Development). The funders had no role in planning, conducting, or interpreting the findings.

## Conflict of Interest

The authors declare that the research was conducted in the absence of any commercial or financial relationships that could be construed as a potential conflict of interest.

## Publisher's Note

All claims expressed in this article are solely those of the authors and do not necessarily represent those of their affiliated organizations, or those of the publisher, the editors and the reviewers. Any product that may be evaluated in this article, or claim that may be made by its manufacturer, is not guaranteed or endorsed by the publisher.
