# Supplementary material for: Short- and Mid-Term Impacts of COVID-19 Outbreak on the Nutritional Quality and Environmental Impact of Diet
Source: Front Nutr. 2022 Mar 11;9:838351. doi: 10.3389/fnut.2022.838351 (PMC8963449; doi:10.3389/fnut.2022.838351)
Supplement: Supplementary file 1 [file Table_1.DOCX]

Supplementary Material

# Supplementary Tables

**Table S1.** Deviations from the pre-registered analytic plan

| **Type of analyses** | **Pre-registered analyses** | **Actual analyses** |
| --- | --- | --- |
| Primary | Linear mixed models will be used to test the effect of time (categorial: before, during, one-year after the lockdown) on the nutrition quality and the environmental impact of the diet, with random effect of participant to account for correlation between repeated measures. We will replicate the linear mixed models described above controlling for age, gender, educational level and declared BMI one year after the first lockdown. | No deviation |
| Secondary (organic and local food consumption) | Linear mixed models will be used to test the effect of time (categorial: before, during, one-year after the lockdown) on the consumption frequency of the 12 categories of organic and locally produced food products, with random effect of participant to account for correlation between repeated measures. We will replicate the linear mixed models described above controlling for age, gender, educational level and declared BMI one year after the first lockdown. | Linear mixed models were run on global organic and local food consumption scores instead of organic and local food consumption scores for each food category. |
| Secondary (food choice motives) | Linear mixed models will be used to test the effect of time (categorial: before, during, one-year after the lockdown) on the nine food choice motives, with random effect of participant to account for correlation between repeated measures. We will replicate the linear mixed models described above controlling for age, gender, educational level and declared BMI one year after the first lockdown. | No deviation. |
| Exploratory | We will examine the influence of short-term and mid-term changes in food choice motives (Δ motives) on short-term and mid -term changes in the nutritional quality (Δ nutritional quality) and environmental impact (Δ GHGE) of diet. Linear mixed models (n=18) will be used including Δ motives (continuous variable), time lag (categorical variable: short-term / mid -term) and Δ motives*time lag as predictors of Δ nutritional quality and Δ environment, with random effect of participant. | To be consistent with what has been done in our previous study investigating the influence of changes in food choice motives during and before the lockdown on the difference in nutritional quality of diet, we ran four multiple linear regressions instead of 18 linear mixed models including the nine Δ motives (short- or mid -term) as predictors and Δ nutritional quality and Δ GHGE as the dependant variables (short- or mid -term). |

**Table S2.** Comparison of participants in the 2020 survey that were and were not included in the 2021 analyses (2020 data)

|  | **Participants included in 2020 analyses only**  **(n=414)** | **Participants included in 2020 and 2021 analyses**  **(n=524)** | ***p*-value*** |
| --- | --- | --- | --- |
| **Participants’ characteristics** |  |  |  |
| **Age,** *years*, mean (SD) | 37.6 (11.1) | 39.5 (12.0) | 0.012 |
| **Gender,** *female*, n (%) | 319 (77.2) | 417 (79.6) | 0.386 |
| **Employment status,** n (%)  *Full or part-time*  *Student*  *Retired*  *Looking for a job*  *Looking after home*  *Other* | 320 (77.3)  32 (7.7)  15 (3.6)  28 (6.8)  7 (1.7)  12 (2.9) | 406 (77.4)  34 (6.5)  33 (6.3)  37 (7.1)  5 (1.0)  9 (1.7) | 0.246 |
| **Highest educational qualification,** n (%)  *< High-school +2 years diploma*  *High-school +2 years diploma*  *High-school +3 or +4 years diploma*  *≥ High-school +5 years diploma* | 98 (21.7)  90 (23.9)  99 (30.7)  127 (23.7) | 129 (24.6)  107 (20.4)  131 (25.0)  157 (30.0) | 0.934 |
| **Dietary restrictions,** *none*, n (%) | 366 (88.4) | 468 (89.3) | 0.660 |
| **Dieting status,** *yes*, n (%) | 58 (14.0) | 74 (14.1) | 0.961 |
| **Reported BMI,** kg/m^2^, mean (SD)  *Implausible weight or height*,* n (%) | 24.7 (4.8)  4 (1.0) | 24.4 (4.9)  6 (1.1) | 0.340 |
| **Dietary outcomes before the first lockdown** |  |  |  |
| Total energy, *kcal/d*, mean (SD)  sPNNS-GS2, mean (SD) | 1742 (613)  1.19 (2.50) | 1667 (581)  1.14 (2.49) | 0.056  0.750 |
| GHGE, *kg CO_2_eq/d*, mean (SD) | 4.96 (2.09) | 4.72 (2.05) | 0.070 |
| GHGE, *kg CO_2_eq/2000 kcal*, mean (SD) | 5.74 (1.63) | 5.67 (1.46) | 0.462 |

*T-tests for continuous variables, Chi-square test for categorical variables
